# Distance-Metric Learning for Personalized Survival Analysis

**DOI:** 10.3390/e25101404

**Published:** 2023-09-30

**Authors:** Wolfgang Galetzka, Bernd Kowall, Cynthia Jusi, Eva-Maria Huessler, Andreas Stang

**Affiliations:** 1 Institute of Medical Informatics, Biometrics and Epidemiology, University Hospital Essen, 45130 Essen, Germany; 2 Nisso Chemical Europe GmbH, 40212 Düsseldorf, Germany

**Keywords:** survival analysis, machine learning, metric learning, kernel regression, personalized medicine

## Abstract

Personalized time-to-event or survival prediction with right-censored outcomes is a pervasive challenge in healthcare research. Although various supervised machine learning methods, such as random survival forests or neural networks, have been adapted to handle such outcomes effectively, they do not provide explanations for their predictions, lacking interpretability. In this paper, an alternative method for survival prediction by weighted nearest neighbors is proposed. Fitting this model to data entails optimizing the weights by learning a metric. An individual prediction of this method can be explained by providing the user with the most influential data points for this prediction, i.e., the closest data points and their weights. The strengths and weaknesses in terms of predictive performance are highlighted on simulated data and an application of the method on two different real-world datasets of breast cancer patients shows its competitiveness with established methods.

## 1. Introduction

One important task in medical research and patient care is accurately predicting clinical outcomes for individual patients. Traditionally, (semi-)parametric approaches have been used, assuming a specific functional relationship, often linear, between the patient’s clinical characteristics and the outcome. However, there is a growing trend towards utilizing supervised machine learning models for this task due to their flexibility and ability to overcome these limitations. Although the focus of this paper is on prediction, machine learning models may not only offer more accurate outcome predictions but also support causal inference. For instance, they can be used to estimate the average treatment effect in the presence of confounding, using targeted maximum likelihood estimation [1]. Another application is the estimation of heterogeneous treatment effects, where the focus is on exploring how the effect of a treatment is influenced by the patient’s clinical characteristics [2,3].

The application of machine learning methods in medical sciences for classification or regression tasks is relatively straightforward. However, when it comes to time-to-event outcomes, which are of central importance in studying chronic diseases that progress over time (such as cancer or cardiovascular diseases), the challenges are more pronounced. This is because standard metrics used for training models, such as log-binomial loss or mean squared error, are not directly applicable due to the presence of (right) censored observations. Right censoring occurs when the true event time is not known for certain individuals, but only a lower bound of it. It can occur due to the loss of follow-up or if the event is not observed until the end of the study period. Despite these challenges, researchers have successfully adapted various machine learning methods [4], such as support vector machines [5], regression trees [6], forests [7], and more recently, deep neural networks [8,9], to handle time-to-event data. Although random survival forests and neural networks, in particular, provide accurate prediction, these models lack interpretability; that is, they do not provide explicit explanations for their predictions. Therefore, they are also referred to as “black box” models [10]. Furthermore, models such as random survival forest still have difficulties in modeling non-proportional hazards.

Instance-based algorithms are a class of machine learning algorithms that provide explicit explanations for individual predictions [11] (Chapter 8). In instance-based learning, the prediction for a new instance (i.e., a data point) is made by comparing it to the instances used for training and calculating weights. These weights are determined using a kernel function and depend on the metric in the covariate space. Training instances that are close to the new instance are assigned higher weights, while those that are far away receive lower weights. The prediction for the new instance is then obtained by taking the weighted average of the outcomes of the training instances. To ensure precise predictions, it is essential to optimize the distance measure and ensure that instances close to each other have similar outcomes. While there are numerous metric learning methods available for classification [12,13] and regression tasks [14], the same is not true for time-to-event outcomes. Existing approaches to time-to-event analysis rely on a weighted Kaplan–Meier estimate for survival prediction, but they have shown mixed results in terms of predictive performance and were not competitive with the random survival forest. Some of these approaches utilize a pre-defined metric [15,16] for calculating the weights, while others discretize the time and learn the metric through maximum likelihood estimation [17].

In this paper, we propose an algorithm that predicts the survival probability based on weighted instances by estimating parameters for the piece-wise exponential distribution. Due to the use of the piece-wise exponential distribution, we are able to model non-proportional hazards. The log-likelihood for continuous distributions is utilized as the loss function to optimize the metric on the covariate space. To minimize the required number of intervals, for an accurate fit of the survival curve’s shape, we employ a global transformation of time. Furthermore, we conduct a comparative analysis of the algorithm’s performance on simulated and real-world data against two widely used approaches: the de facto standard, the Cox proportional hazards model, and the easily applicable and widely adopted random survival forest.

The structure of the remaining paper is as follows: In Section 2.1, we formally introduce the problem and provide a clear definition. We briefly sketch the well-established Cox proportional hazards and random survival forest approaches in Section 2.2. In Section 2.3 and Section 2.4, we present our approach, the kernel survival prediction with piecewise exponential distributions. Moving on to the experimental setup, in Section 3.1, we outline the conducted experiments and describe the performance measures utilized. We also discuss the distributions used for simulating data in Section 3.2. In Section 4, we present the results obtained from the conducted experiments. Finally, in the concluding Section 5, we summarize our findings and provide an outlook for future research directions.

## 2. Materials and Methods

### 2.1. Problem Setting

By capital letters X,Y,C, we denote the random variables for the covariates, the time until an event, and the time until censoring respectively. Of those, only realizations of *X* are observed. Instead of *Y* and *C*, the time T=min(Y,C) until event or censoring, whichever occurs first, and Δ=1(Y≤C), an indicator which is 1 if an event occurred before censoring and 0 otherwise, are observed. Our aim is to estimate the conditional survival probability

$$\begin{array}{c} S(t|x):=P(Y>t|X=x), \end{array}$$

given i.i.d. realizations $D=\left(x_{1},t_{1},\delta_{1}\right),\ldots ,\left(x_{n},t_{n},\delta_{n}\right)\in R^{m}\times R_{>0}\times \left\{0,1\right\}$ of X,T and Δ. Equivalently, we can estimate the hazard rate λ(t|x) or the cumulative hazard Λ(t|x), which are given by

(1) $$\begin{array}{cc} \lambda (t|x) & :=\lim_{\epsilon \to 0}\frac{S(t|x)-S(t+\epsilon |x)}{\epsilon S(t|x)},\;\mathrm{and}\\ \Lambda (t|x) & :=\int_{0}^{t}\lambda \left(t^{\prime }|x\right)dt^{\prime }. \end{array}$$

The survival function can be retrieved from the cumulative hazard through S(t|x)=exp(−Λ(t|x)). The hazard rate at time *t* can be interpreted as the risk of an event in the next instant, given no event occurred until *t*. Oftentimes it is more convenient to model the hazard λ instead of modelling *S* directly. Throughout, we assume that *C* and *Y* are independent given *X*. Thus [18], the likelihood of an observation (x,t,δ) satisfies

(2) $$\begin{array}{c} f\left(T=t,\Delta =\delta |X=x\right)\propto \lambda {\left(t|x\right)}^{\delta }\exp\left(-\Lambda \left(t|x\right)\right). \end{array}$$

### 2.2. Survival Prediction Based on Proportional Hazards

In this subsection, we quickly review the Cox regression and the idea behind the splitting rule for trees used to build the random survival forest. For the conventional Cox regression model, one assumes that the hazard rate λ(t|x) can be factorized in one term only depending on *t*, the so-called baseline hazard $\lambda_{0}$, and one term only depending on *x*, i.e.,

$$\begin{array}{c} \lambda \left(t|x\right)=\lambda_{0}\left(t\right)\exp\left(h\left(x\right)\right). \end{array}$$

Furthermore, one assumes that *h* is linear in *x*, i.e., $h\left(x\right)=h_{\beta }\left(x\right)=\sum_{i=1}^{m}\beta_{i}x_{i}$ for some coefficients $\beta_{i}\in R$. Under this assumption, we have

$$\begin{array}{c} \frac{\lambda (t|x_{1})}{\lambda (t|x_{2})}=\frac{\exp(h\left(x_{1}\right))}{\exp(h\left(x_{2}\right))}, \end{array}$$

i.e., the hazard rates of $x_{1}$ and $x_{2}$ are proportional to each other independent of time *t*. One can show that the likelihood can be maximized with respect to β independently of λ(t), by maximizing the partial likelihood

$$\begin{array}{c} L_{C}=\prod_{i|\delta_{i}=1}\frac{\exp(h_{\beta }\left(x_{i}\right))}{\sum_{j|t_{j}\geq t_{i}}\exp\left(h_{\beta }\left(x_{j}\right)\right)}. \end{array}$$

To obtain survival predictions from the Cox regression model, it is then necessary to calculate the cumulative baseline hazard λ(t) using the Breslow estimator. Instead of using a linear *h* it is also possible to use more complex structures like deep neural networks [8].

The partial likelihood $L_{C}$ can also be used to derive a splitting criterion for random survival trees. To this end, each split gives rise to a binary covariate indicating which child node an observation is assigned to. Calculating the minimal partial likelihood $L_{C}$ with respect to that covariate is a measure of the predictive quality of the split and, hence, can be used to pick the best split. This is, in fact, the basic idea of the splitting rule used for the most common random survival forests [7,19]. To estimate the cumulative hazard or survival probability within each leaf, one can utilize either the Nelson–Aalen estimate or the Kaplan–Meier estimate for the instances contained in the leaf.

One can expect that for both methods, the Cox regression model and the random survival forest, predictive performance decreases with increasing non-proportionality of the hazards.

### 2.3. Survival Prediction with Kernels

Predicting the survival probabilities for a patient with covariates *x*, based on the training data ${\left(x_{i},t_{i},\delta_{i}\right)}_{i=1,\ldots ,n}$, involves two steps. Firstly, we assign weights $w_{i}$ to each instance in the training set, quantifying the similarity between *x* and $x_{i}$. Secondly, we utilize the weighted training data to estimate the parameters of a survival distribution for *x*.

The notion of similarity in the covariate space is formalized through a kernel function, denoted as $k:R^{m}\times R^{m}\to R$. A kernel function is a symmetric function which satisfies that the matrix $k{\left({\tilde{x}}_{i},{\tilde{x}}_{j}\right)}_{i,j=1,\ldots ,l}$ is positive semi-definite for any choice of l∈N and ${\tilde{x}}_{1},\ldots ,{\tilde{x}}_{l}\in R^{m}$. Although a variety of different kernel functions exists [20], we have for simplicity chosen to focus on the radial basis function. Consequently, the weights $w_{i}$ in our case are calculated as

$$\begin{array}{c} w_{i}=\exp\left(-d^{2}\left(x,x_{i}\right)\right), \end{array}$$

where $d:R^{m}\times R^{m}\to R_{\geq 0}$ is a (pseudo)metric. For our algorithm, fitting entails learning *d*. We confine ourselves to the subset of metrics where the distance is induced by an inner product, meaning that we have

$$\begin{array}{c} d^{2}\left(x,x_{i}\right)={\left(x-x_{i}\right)}^{⊤}A\left(x-x_{i}\right), \end{array}$$

for a positive semi-definite, symmetric matrix *A*. Using the Cholesky decomposition, we have $A=U^{⊤}U$ for an upper triangular matrix *U*. Hence, we can simply learn *U* instead of *A* and the expression for the weights $w_{i}$ simplifies to $w_{i}\left(U,x\right)=\exp\left(-{∥Ux-Ux_{i}∥}^{2}\right)$, where ∥·∥ denotes the Euclidean norm. This first step is common in instance-based learning and used for regression tasks [14] as well as for survival prediction [15,17].

What mainly distinguishes our approach from existing methods, in regard to learning a metric for survival prediction, is the second step, i.e., the actual prediction of the survival probabilities using ${\left(w_{i}\left(U,x\right),t_{i},\delta_{i}\right)}_{i=1,\ldots ,n}$, and the weighted outcomes of the given training data D. While in existing approaches the survival predictions are derived from the weighted outcomes using the non-parametric weighted Kaplan–Meier estimate, we use the weighted outcomes to estimate the parameters of a piece-wise exponential distribution. To this end, it is necessary to split the time into k∈N disjoint intervals, i.e., $R_{>0}=\left(\tau_{0},\tau_{1}\right]\cup \left(\tau_{1},\tau_{2}\right]\cup \ldots \cup \left(\tau_{k-1},\tau_{k}\right)$, with $\tau_{0}=0,\tau_{k}=\infty$ and *k* being a chosen number. Denote by $\delta_{ij}:=\delta_{i}1\left(t_{i}\in \left(\tau_{j-1},\tau_{j}\right]\right)$ if for the *i*-th training instance the event occurred in the *j*-th interval, by $r_{j}\left(t\right):=\left|\left(0,t\right]\cap \left(\tau_{j-1},\tau_{j}\right]\right|$ the time at risk in interval *j* for an observed time *t* and, furthermore, by j(t) the index of the interval containing *t*. The hazard rate in the *j*-th interval, as the maximum likelihood estimate of the weighted outcomes under the piece-wise exponential distribution, is then given by

$$\begin{array}{c} \lambda_{j}\left(x\right)=\frac{\sum_{i=1}^{n}w_{i}\left(U,x\right)\delta_{ij}}{\sum_{i=1}^{n}w_{i}\left(U,x\right)r_{j}\left(t_{i}\right)}. \end{array}$$

Its derivation can be found in Appendix A. Using (1), we obtain

$$\begin{array}{cc} \Lambda (t|x) & =\sum_{j=1}^{k}\lambda_{j}\left(x\right)r_{j}\left(t\right)\;\mathrm{and}\\ S(t|x) & =\exp(-\sum_{j=1}^{k}\lambda_{j}\left(x\right)r_{j}\left(t\right)), \end{array}$$

for the cumulative hazard and the survival probability. While within the intervals the hazard rates, and therefore the hazard ratios, are constant, they will in general differ across intervals, i.e., $\lambda_{j}\left(x\right)/\lambda_{j}\left(\tilde{x}\right)\neq \lambda_{l}\left(x\right)/\lambda_{l}\left(\tilde{x}\right)$, for j≠l.

Let us now finally describe how to obtain the metric-inducing transformation *U*. We obtain the log-likelihood for an observation (x,t,δ) under the model by plugging the above equation in (2). Hence, we obtain

$$\begin{array}{c} \log\left(f\left(T=t,\Delta =\delta |x\right)\right)=\delta \log\left(\frac{\sum_{i=1}^{n}w_{i}\left(U,x\right)\delta_{ij(t)}}{\sum_{i=1}^{n}w_{i}\left(U,x\right)r_{j(t)}\left(t_{i}\right)}\right)-\sum_{j=1}^{k}\frac{\sum_{i=1}^{n}w_{i}\left(U,x\right)\delta_{ij}}{\sum_{i=1}^{n}w_{i}\left(U,x\right)r_{j}\left(t_{i}\right)}r_{j}\left(t\right), \end{array}$$

where we adopted the convention 0log0=0. However, instead of obtaining *U* by maximization of the log-likelihood on the training data, we add a regularization term $\eta {∥U∥}^{2}$, with η>0 being another hyperparameter, and, as in Weinberger and Tesauro [14], set $w_{i}\left(U,x_{i}\right)=0$, both to prevent overfitting. More specifically, by setting $w_{i}\left(U,x_{i}\right)=0$ we make sure that, for learning *U*, the *i*-th instance is not used to predict its own outcome. Adding the term $\eta {∥U∥}^{2}$ results in a smaller *U*, which decreases the variance of the weights. Other regularization schemes, such as the more general elastic net regularization, could also be used [21]. All in all, *U* is then given by

$$\begin{array}{c} U=\underset{\tilde{U}}{\mathrm{argmin}}\left(\eta {∥\tilde{U}∥}^{2}+\frac{1}{n}\sum_{l=1}^{n}\left(\delta_{l}\log\frac{\sum_{i=1}^{n}w_{i}\left(\tilde{U},x_{l}\right)\delta_{ij(t_{l})}}{\sum_{i=1}^{n}w_{i}\left(\tilde{U},x_{l}\right)r_{j(t_{l})}\left(t_{i}\right)}-\sum_{j=1}^{k}\frac{\sum_{i=1}^{n}w_{i}\left(\tilde{U},x_{l}\right)\delta_{ij}}{\sum_{i=1}^{n}w_{i}\left(\tilde{U},x_{l}\right)r_{j}\left(t_{i}\right)}r_{j}\left(t_{l}\right)\right)\right) \end{array}$$

We note that the computational complexity of the loss grows quadratically with the number of training samples, which might make our approach unpractical for large datasets and may require adaptations. Additionally, it is important to acknowledge the non-convex nature of the loss function, which may necessitate multiple random initializations of the matrix *U* when utilizing a gradient-based optimizer.

Furthermore, let us consider the available hyperparameters. In this paper, we solely focus on the Gaussian radial basis function as the kernel function, but other options exist. Moreover, the number and location of time intervals for the piece-wise exponential distribution have to be picked. Through our experiments, we will demonstrate that typically only a few (≤5) intervals are needed for the algorithm to perform well when using the time transformation introduced in the next subsection. Lastly, the regularization parameter, denoted by η, has to be selected.

### 2.4. Transformation of Time

The shape of the survival curve might be very different from an exponential distribution, and thus to appropriately model the survival we would need a high number of time intervals. To avoid this issue, we use a time transformation introduced in LeBlanc and Crowley [22]. This transformation, denoted as $\varphi :t\to \tilde{t}$, is designed such that the transformed times for observations with events correspond to the Nelson–Aalen estimates of the cumulative hazard. For censored observations, the transformed times are linearly interpolated. This approach ensures that the survival prediction of the (unweighted) exponential model on the transformed times coincides with those of the Nelson–Aalen model.

By using this transformation, the need for a high number of time intervals is reduced, as the different time intervals are only required to model non-proportional hazards.

## 3. Experiments on Simulated and Real-World Data

### 3.1. Description of Data and Evaluation Criteria

We conducted a performance evaluation of the kernel prediction method by comparing it against two established approaches in survival analysis on simulated and real-world datasets: Cox regression, which serves as the de facto standard, and survival forest, which is widely used and easily applicable.

For the simulated data, we varied several parameters, such as the training data sizes, the degree of interaction, the nonlinear rescaling of covariates, the non-proportionality of hazards, and the proportion of censoring to evaluate the effect on the predictive performance. Training and evaluation data were drawn from the same distributions. For each setting, we ran 100 simulations.

In addition to the simulated data, we also evaluated the performance of our method on two publicly available datasets: the METABRIC dataset [8,23] (1904 observations, 9 covariates, 42% censoring) and the Rotterdam/GBSG dataset [8,24,25] (2232 observations, 7 covariates, 43% censoring). Both datasets contain clinical attributes and information on survival of breast cancer patients. To obtain an unbiased assessment, we partitioned each dataset into randomly selected separate training and test sets. To investigate the influence of training data size on performance, we varied the size of the training set, including proportions of 30%, 50%, and 70% of the total data. We considered 100 different splits for each training data size to account for random variation and obtain reliable performance estimates. Unlike the random survival forest, the Cox regression and the introduced kernel prediction are not invariant under monotone transformations of the numerical covariates. Hence, to illustrate the importance of the choice of the appropriate scale on real-world data, we performed an additional analysis on the Rotterdam/GBSG dataset where we replaced the two covariates indicating levels of progesterone and estrogen, given in fmol/mg, by their logarithm, as this is a more natural scale for concentrations.

Antolini’s concordance index [26] and the integrated Brier score [27] were employed as performance measures. The concordance index was utilized to evaluate the discriminative performance. To calculate Antolini’s concordance index, one examines all pairs within the test set that can be ordered, meaning we can determine which event occurs first. This is the case if an event took place at the earlier event time, i.e., this set consists of all pairs i,j=1,…,n which satisfy the conditions $t_{i}<t_{j}$ and $\delta_{i}$ = 1. The concordance index is the proportion of those pairs which are ordered correctly by the model in the sense that $\hat{S}\left(t_{i}|x_{i}\right)<\hat{S}\left(t_{i}|x_{j}\right)$. Hence, the concordance index is an estimate for

$$\begin{array}{c} P(S\left(T_{i}|X_{i}\right)<S\left(T_{i}|X_{j}\right)|T_{i}<T_{j}\;\mathrm{and}\;\Delta_{i}=1), \end{array}$$

for a randomly chosen pair i,j of subjects. The higher the concordance index the better. A concordance index of 0.5 indicates that the predictions are not informative in terms of discrimination. The integrated Brier score on the other hand primarily assesses calibration. The Brier score at a specific time point is calculated as the weighted mean of squared differences between the predicted survival probability and the corresponding state (1 for alive, 0 for deceased) of an instance at that time. The inverse probability of censoring weighting is employed as a weighting scheme, ensuring that the Brier score for time *t* provides an unbiased estimate of

$$\begin{array}{c} E\left[{\left(1\left(Y>t\right)-S\left(t|X\right)\right)}^{2}\right]. \end{array}$$

To obtain the integrated Brier score, a single performance index for the whole follow-up, the Brier score is integrated numerically up to a specified time, which we chose to be the 95% quantile of the event times. As the final evaluation measure for a model *M*, we do not use the integrated Brier score $B_{I}\left(M\right)$ directly, but the improvement with respect to the Kaplan–Meier estimator, $R^{2}:=1-B_{I}\left(M\right)/B_{I}\left(KM\right)$, as suggested in Graf et al. [27] to measure the explained residual variation. While these two criteria are the most common to evaluate model performance [4], further model measures and diagnostics, such as graphical analysis of calibration [28], exist.

The experiments were carried out in Python 9.3.16. The considered hyperparameters for the methods were optimized using grid search and 10-fold cross-validation on the training data. The implementation in [19] of the random survival forest was used. The number of features used per split and the minimal number of observations per node were optimized. The classical Cox regression, with the linear dependency of the log hazard on the covariates, was carried out with [29]. For the kernel prediction, we only determined the regularization parameter η by cross-validation, the number of time intervals was set to four for all experiments. The quantiles of the observed event times were chosen as splitting times to ensure a sufficient number of events in each time interval. Optimization of the log-likelihood was carried out with LBFGS [30] implemented in PyTorch 1.12.1 [31] on the standardized predictors with the identity matrix as the initial value. More details on the considered hyperparameters can be found in Appendix B.

### 3.2. Description of Simulation Settings

We conducted four series of simulations. In the first, series A, we varied the degree of interactions in a controlled way, in the second, series B, we varied the degree of non-proportionality of hazards, in the third, series C, we varied a monotonic transform on the covariates, and in the fourth, series D, we studied the behavior under ties. In all those series of simulations, the covariates *X* were drawn from an m−dimensional standard normal distribution. The parameter μ, specifying the location (series A, C and D) or log-scale (series B) of the survival distribution, depended in all simulations on the covariates *X* via $\mu \left(X\right)=\alpha_{q}\left(X^{⊤}MX-E_{X}\left[X^{⊤}MX\right]\right)+\alpha_{\ell }X^{⊤}b$. The matrix $M\in {\mathrm{Sym}}_{m}\left(R\right)$ and the vector $b\in R^{m}$ were randomly chosen at each run of the simulation with $b_{i},$$M_{ij}$∼N(0,1) for i=1,…,m,j≠i, and $M_{ii}=0$. Thus, $X^{⊤}MX$ specifies the dependency of μ on interaction terms of the covariates, while $X^{⊤}b$ specifies the linear dependency. Noting that ${\mathrm{Var}}_{X}\left(\mu_{X}\right)=\alpha_{q}^{2}2\mathrm{Tr}\left(M^{2}\right)+\alpha_{\ell }b^{⊤}b$, we chose the parameters $\alpha_{q}$ and $\alpha_{\ell }$ in a controlled way, such that (i) ${\mathrm{Var}}_{X}\left(\mu \left(X\right)\right)=1$ and (ii) a specified percentage of the variance of μ stemmed from the interaction terms $X^{⊤}MX$.

Simulation series A generated survival data by assuming a fixed shape of the survival curve, with the survival time *Y* following a lognormal distribution with log(Y)∼N(μ,1). The parameters $\alpha_{q},\alpha_{\ell }$ were varied, such that either 0, 25, 50, 75, or 100% of the variance of μ stemmed from the interaction terms.

Simulation series B examined the performance of the models when one covariate breaks the proportional hazards assumption by influencing the shape of the survival curve. In this simulation, the event times were drawn from a Weibull distribution, i.e., *Y*∼Wei(Scale,Shape). To break the proportional hazards assumption, an additional binary covariate $X_{m+1}$ with $P\left(X_{m+1}=1\right)=P\left(X_{m+1}=0\right)=0.5$ specifying the shape of the curve was introduced. Specifically, we have

$$\begin{array}{c} Y∼\left\{\begin{array}{c} \mathrm{Wei}\left(\exp\left(\mu \left(X\right)\right),\theta_{0}\right),\mathrm{for}\;X_{m+1}=0,\\ \mathrm{Wei}\left(\exp\left(\mu \left(X\right)+\alpha_{1}\right),\theta_{1}\right),\mathrm{for}\;X_{m+1}=1. \end{array}\right. \end{array}$$

The parameters $\alpha_{1}$, $\theta_{0}$, and $\theta_{1}$ were varied such that (i) the hazard ratio between the survival curves $\mathrm{Wei}(1,\theta_{1})$ and $\mathrm{Wei}(\exp\left(\alpha_{1}\right),\theta_{1})$ was approximately 1 and (ii) the integrated squared difference between the two curves varied from 0 to 0.3 in approximately equidistant steps. In simulation series B, $\alpha_{q}$ and $\alpha_{\ell }$ were chosen, such that 50% of the variance of μ was explained by the interaction terms. The exact values of $\alpha_{1}$, $\theta_{0}$, and $\theta_{1}$ and a plot of the survival curves for μ=0 can be found in Appendix C.

For simulation series C and D, we used the same survival distribution as in A and again fixed $\alpha_{q},\alpha_{\ell }$, such that 50% of the variance of μ came from the interaction terms. However, for the simulations of series C, we did not use the realizations of *X* for fitting the prediction models but their values under the pointwise transformation $x\mapsto {\mathrm{sgn}\left(x\right)|x|}^{p}$ with p∈{0.5,0.75,1,1.25,1.5,1.75,2}. For series D of simulations, we discretized the observed times of the training set by mapping them to the median of those quantiles of the event times that contained them. The different observed times in the training data varied from 15 over 30 and 60 up to 120.

For all four series of simulations, censoring times were generated from a Weibull distribution with a shape parameter of 1.2. The scale of this distribution was varied, such that either 0%, approximately 25%, or 50% of observations were censored. To reduce variance and to allow for a better understanding of performance loss due to the censoring of training data, the predictions of the models were evaluated against the uncensored test data. In all simulations, we set m=6 and drew, for each run, 400 samples for the training set and 4000 samples for the test set. We also conducted a sensitivity analysis to see if our results depended on the size of the training data and conducted the same series of analyses with a training set of 200 samples.

## 4. Results

### 4.1. Results of Simulation

**Figure 1:**
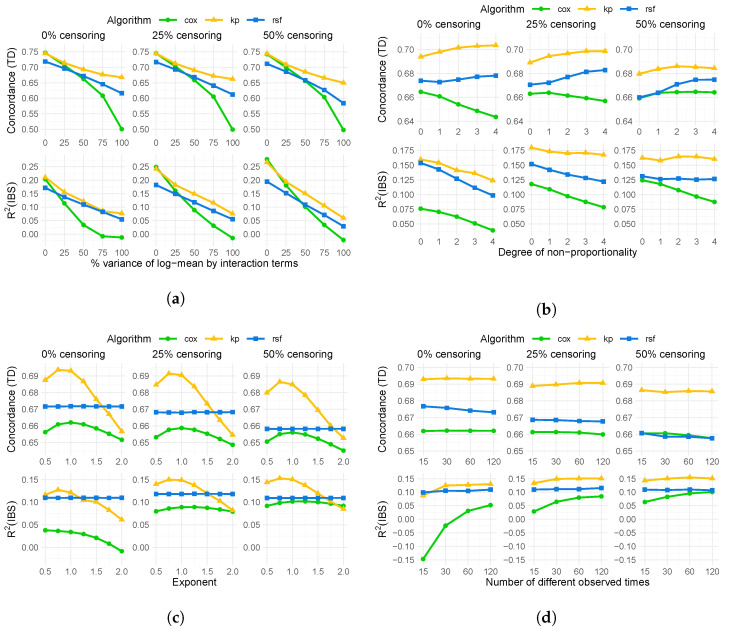
Mean concordance index and mean $R^{2}$ with respect to the integrated Brier score (IBS) of Cox proportional hazards model (Cox), kernel prediction (kp), and random survival forest (rsf) over 100 runs under different settings with a training size of n=400. (**a**) Simulation A: By degrees of interaction. (**b**) Simulation B: By non-proportionality of hazards. (**c**) Simulation C: By exponent of covariate transformation. (**d**) Simulation D: By different numbers of observed times.

The results of simulation A are shown in Figure 1a. We see that the concordance and the $R^{2}$ decrease for all models as the level of interaction increases. The decrease in performance is almost parallel for the random survival forest and the kernel prediction, with the kernel prediction being better. If there is no interaction at all, the Cox model performs slightly superior than the other models as its assumptions are approximately met. However, the performance declines rapidly with increasing levels of interactions, eventually becoming uninformative. While the performance of the Cox model could be improved by adding interaction terms, the simulation highlights an advantage of machine learning models as they do not require the specification of a certain functional form between covariates and outcomes. Moreover, as the censoring rate increases, the relative performance of the kernel prediction compared to the random survival forest increases slightly.

The results of simulation B are displayed in Figure 1b. For this simulation, the result strongly depends on the level of censoring, as the censoring influences the hazard ratio as well as the log-rank score [32]. Regarding concordance, we see that, except for the Cox proportional hazards model, the performance increases with the non-proportionality of hazards regardless of censoring. For the Cox model, the concordance decreases drastically when there is no censoring, but remains relatively constant when the censoring is 25 or 50%. The $R^{2}$ decreases for all models when censoring is absent or low. However, the kernel prediction shows by far the lowest decrease. When there is 50% censoring, only the Cox model shows a decrease in performance, while the performance of the other methods remains relatively constant.

The results of simulation C can be found in Figure 1c. The performance of the random forest is the same for all transformations, as for the algorithm only the ordering of the covariates is important. The kernel prediction, on the other hand, shows a very high dependence on the nonlinear scaling in terms of both, concordance and $R^{2}$. Depending on the transformation, its performance varies from being the best algorithm to a performance similar to the Cox model, below the random survival forest. The performance of the Cox model depends on the nonlinear scaling as well, although it is less sensitive than the kernel prediction. Also in this series of simulations, the relative performance of the kernel prediction compared to the random survival forest seems to increase with increasing censoring rate.

Lastly, the results of simulation D can be found in Figure 1d. Performance, in terms of concordance index and $R^{2}$, remains quite stable for kernel prediction and random survival forest. For the Cox regression model, we see that while the concordance index remains stable, the $R^{2}$ decreases when we only have a few different observed times, i.e., when the number of ties is high. The decrease is more pronounced when censoring is absent or small.

The results of sensitivity analysis for a smaller training set of size n=200 showed the same patterns and can be found in Appendix D.

Overall we see similar trends in the performances of kernel prediction and random survival forest when the level of interaction increases. The kernel prediction handles non-proportional hazards better, while it is, unlike the random survival forest, very sensitive to nonlinear scaling of the covariates. If the scaling is suitable though, it consistently outperforms the random survival forest. Of the examined algorithms, the Cox model depends on most assumptions and its performance decreases fast in all considered scenarios when those assumptions are not met.

### 4.2. Results on Real-World Data

**Figure 2:**
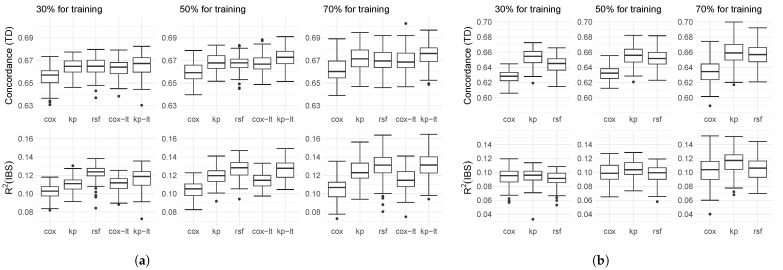
Box plot of concordance index and $R^{2}$ with respect to the integrated Brier score (IBS) from 100 different train/test splits for the Cox proportional hazards model (Cox), kernel prediction (kp), and random survival forest (rsf). For Rotterdam/GBSG additionally with log-transform (lt) of two covariates. (**a**) Performance on Rotterdam/GBSG. (**b**) Performance on METABRIC.

The box plots of the performance on real-world data are shown in Figure 2. In the case of the Rotterdam/GBSG dataset, kernel prediction and random survival forest show similar performance in terms of concordance across all training and test sizes. However, when it comes to $R^{2}$, random survival forest outperforms the other methods, while the Cox regression model performs the worst. It is worth noting that as the training size increases, the performance of kernel prediction catches up with the random survival forest. One reason for the comparable poor performance of the Cox regression model and the kernel prediction is the inappropriate scaling of the numerical variables indicating the concentration of progesterone and estrogen. For the rescaled data, we see that just like for simulated data and the METABRIC dataset, kernel prediction outperforms the random survival forest in terms of concordance. The $R^{2}$ is slightly better for the random survival forest for smaller training sizes, but almost identical when we use 70% of the data for training. Also, the Cox regression model performs substantially better after rescaling of the covariates. An example of how similar patients can be used to explain individual predictions on the Rotterdam/GBSG dataset is in Appendix F.

**Figure 3:**
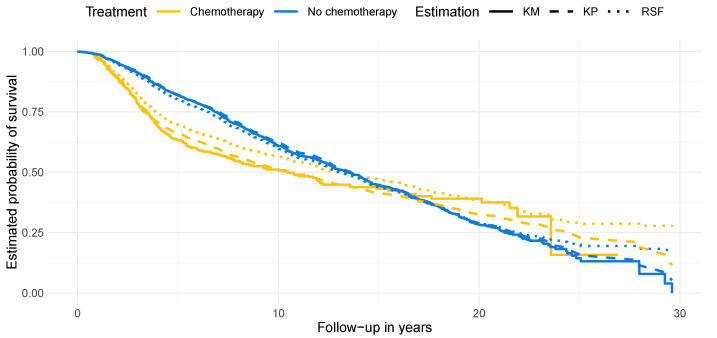
Survival probabilities of patients from the METABRIC dataset stratified by treatment with chemotherapy estimated with the Kaplan–Meier method (KM), kernel prediction (KP), and random survival forest (RSF).

The results for the METABRIC dataset are shown in Figure 2b. We see that when using a small training set, kernel prediction demonstrates superior performance in terms of concordance while the $R^{2}$ is similar for all methods. As the training size increases, the performances of kernel prediction and random survival forest become similar in terms of concordance but kernel prediction becomes superior in terms of $R^{2}$. We assume that part of the superior performance for the METABRIC dataset is due to the better ability of the kernel prediction to handle non-proportional hazards. Figure 3 illustrates this assumption by presenting the Kaplan–Meier estimates for two distinct subgroups within the dataset: the patients who received chemotherapy (*n* = 396) and those who did not (*n* = 1508). The Kaplan–Meier curves intersect with each other, indicating a violation of the proportional hazards assumption. Additionally, we depict the mean survival probability for each subgroup estimated by kernel prediction and random survival forest, which ideally should coincide with the Kaplan–Meier curves. The estimate of the Cox proportional hazard model, where it is not possible for the curves of the different treatment groups to intersect, can be found in Appendix E. It can be seen that both algorithms, the kernel prediction and the random survival forest, estimate the average survival probabilities of the patients without chemotherapy, the larger subgroup, well. For the treated patients, however, we see that the random survival forest produces highly biased survival estimates, which strongly overestimate the survival probabilities throughout the entire 30-year follow-up period. The kernel prediction, on the other hand, is quite accurate up to year 15, particularly when there is still a considerable number of patients at risk. For the remaining years, from year 15 to 30, it looks like a smoothed version of the Kaplan–Meier estimate, as it replaces the step-wise drops with a continuous decrease.

## 5. Discussion

In this paper, we introduced a kernel learning approach for survival analysis that is easy to implement and provides direct interpretability of individual predictions through nearest neighbors. Our method proved to be competitive with other easily applicable techniques, such as random survival forest, on simulated and real-world data. However, there are still some limitations, open problems, and avenues for future research.

One drawback of our proposed kernel prediction compared to the random survival forest is that the latter can deal with missing values more effectively, e.g., via surrogate splits. Another limitation is the sensitivity of the proposed algorithm to the nonlinear scaling of covariates. It would be worth exploring the possibilities of learning a suitable scaling in a data-driven fashion to improve the predictive performance of the kernel prediction. When scaling of covariates is appropriate, kernel prediction performs better than random survival forest in our simulations and on the used real-world data.

Although the paper already demonstrates competitiveness in terms of predictive accuracy when considering only a single hyperparameter, namely the regularization, there are further hyperparameters that can be considered, such as the number and position of the time intervals. While for the experiments in this article, we did not vary the number of intervals, and used the quantiles to position them; this choice was made ad hoc and might not be ideal, in particular when event times are clustered. An investigation on how to best pick the intervals would also greatly benefit other methods using a piece-wise approach, such as neural networks [9]. In addition, the impact of the choice of the kernel function and the norm used in it can be explored. We solely focused on the Gaussian radial basis function; however, many other different possibilities exist. Apart from choosing one kernel, one might also consider employing algorithms for multiple kernel learning to best combine different kernels [33].

As already mentioned, the computational complexity of the presented algorithm scales quadratically in the number of observations, which limits the application to small- to medium-sized datasets. One ad hoc solution is to only use a subset of neighbors, e.g., the closest 1000 for each neighbor, for the calculation and optimization of the kernel, as suggested in [14]. Another possibility is to leverage kernel approximation methods, such as random features [34] or sparse grids for kernel learning [35]. These approaches could help reduce computational complexity while maintaining acceptable predictive performance.

Looking beyond the specific application of survival prediction, our kernel learning approach has a broader use. By deriving a meaningful metric between patients based on the learned kernel, our method enables clinicians to query for similar patients or facilitate subgroup discovery through metric-based clustering. This broader context opens up new avenues for utilizing our method in personalized medicine and healthcare applications.

## Figures and Tables

**Figure A1:**
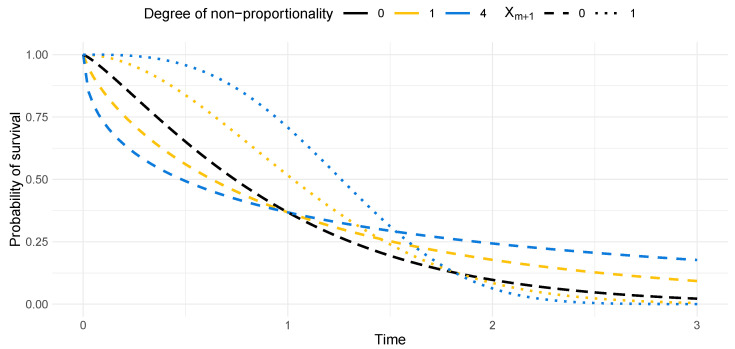
Survival probabilities in simulation B for μ=0 depending on $X_{m+1}$ for the selected degrees of non-proportionality. If the degree of non-proportionality is 0, $X_{m+1}$ does not have an effect on the shape of the curve.

**Figure A2:**
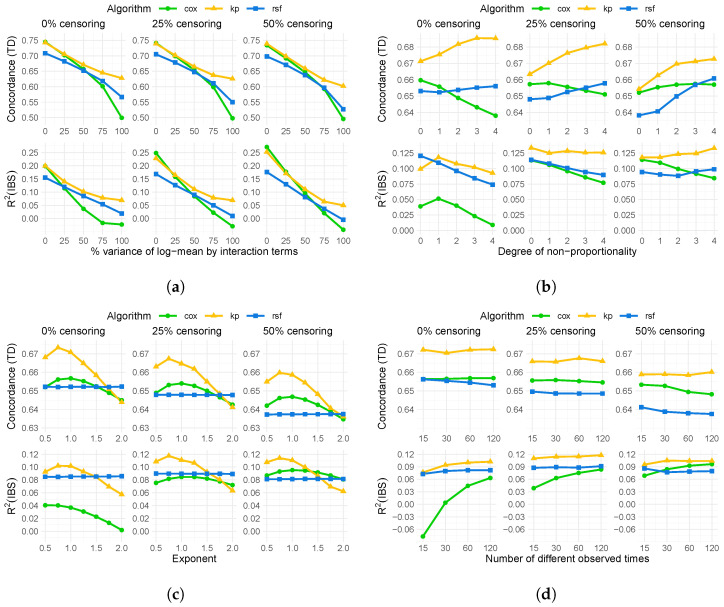
Mean concordance index and mean $R^{2}$ with respect to the integrated Brier score (IBS) of the Cox proportional hazards model (Cox), kernel prediction (kp), and random survival forest (rsf) under different settings and with a training size of n=200. (**a**) Simulation A: By degrees of interaction. (**b**) Simulation B: By non-proportionality of hazards. (**c**) Simulation C: By exponent of covariate transformation. (**d**) Simulation D: By different numbers of observed times.

**Figure A3:**
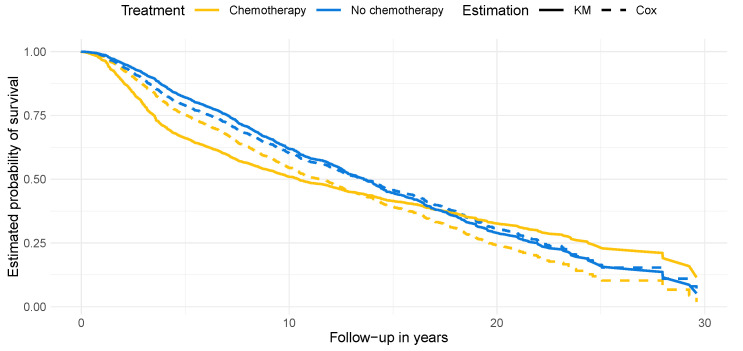
Survival probabilities of patients from the METABRIC dataset, stratified by treatment with chemotherapy, estimated with the Kaplan–Meier method (KM) and by the Cox proportional hazards model (Cox).

**Figure A4:**
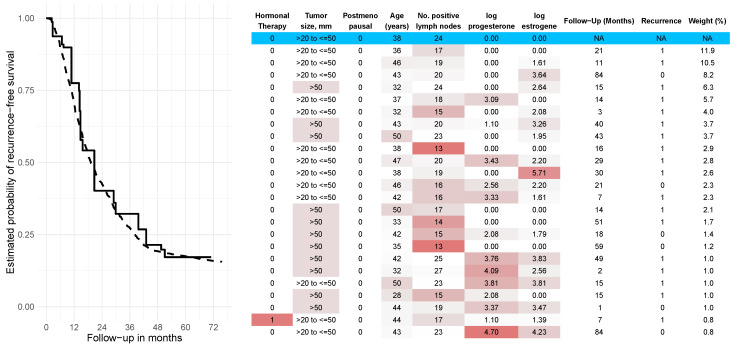
Example of an explanation of a kernel prediction via nearest neighbors on the Rotterdam/GBSG dataset. The table shows the clinical characteristics of the patient for whom we predict recurrence-free survival (first-line, blue background) and of the most important patients used for prediction, ranked by the assigned weights (right column). Attributes that cause dissimilarity according to the learned metric are marked in red. The left side shows the weighted Kaplan–Meier curve (solid line) and the smooth kernel prediction (dashed line).

## Data Availability

Publicly available datasets were analyzed in this study. This data can be found here: https://github.com/jaredleekatzman/DeepSurv/tree/master/experiments/data. The python code to generate the simulated data is available upon request.

## Appendix A

Given the weighted outcomes $\left(w_{1},t_{1},\delta_{1}\right),\ldots ,\left(w_{n},t_{n},\delta_{n}\right)$, the log-likelihood of a single observation $\left(w_{i},t_{i},\delta_{i}\right)$, assuming a piece-wise constant hazard rate $\lambda \left(t\right)=\sum_{l=1}^{k}\lambda_{l}1\left(t\in \left(\tau_{l-1},\tau_{l}\right]\right)$ is, using (2), given by

$$\begin{array}{cc} \log f(T=t_{i},\Delta =\delta_{i}|\lambda_{l=1,\ldots ,k}) & =\log\left(\lambda_{j(t_{i})}^{\delta_{i}}\exp\left(-\int_{0}^{t_{i}}\sum_{l=1}^{k}\lambda_{l}1\left(t^{\prime }\in \left(\tau_{l-1},\tau_{l}\right]\right)dt^{\prime }\right)\right)+\mathrm{Const}.\\ \; & =\delta_{i}\log\lambda_{j(t_{i})}-\sum_{l=1}^{k}\lambda_{l}r_{l}\left(t_{i}\right)+\mathrm{Const}.\\ \; & =\sum_{l=1}^{k}\left(\delta_{il}\log\lambda_{l}-\lambda_{l}r_{l}\left(t_{i}\right)\right)+\mathrm{Const}., \end{array}$$

where the constant only depends on the censoring distribution. Hence, for the full-weighted log-likelihood, we obtain

$$\begin{array}{c} \log f\left({\left(w_{i},T=t_{i},\Delta =\delta_{i}\right)}_{i=1,\ldots ,n}|\lambda_{l=1,\ldots ,k}\right)=\sum_{i=1}^{n}w_{i}\sum_{l=1}^{k}\left(\delta_{il}\log\lambda_{l}-\lambda_{l}r_{l}\left(t_{i}\right)\right)+\mathrm{Const}. \end{array}$$

For j=1,…,l, the maximum likelihood estimate satisfies

$$\begin{array}{cc} \; & 0=\frac{\partial }{\partial \lambda_{j}}\log f\left({\left(w_{i},T=t_{i},\Delta =\delta_{i}\right)}_{i=1,\ldots ,n}|\lambda_{l=1,\ldots ,k}\right)\\ \; & \;\;\;=\sum_{i=1}^{n}w_{i}\sum_{l=1}^{k}\left(\delta_{il}\frac{1}{\lambda_{l}}1\left(l=j\right)-1\left(l=j\right)r_{l}\left(t_{i}\right)\right)\\ \; & \;\;\;=\sum_{i=1}^{n}w_{i}\left(\delta_{ij}\frac{1}{\lambda_{j}}-r_{j}\left(t_{i}\right)\right)\\ \; & ⟹\lambda_{j}=\frac{\sum_{i=1}^{n}w_{i}\delta_{ij}}{\sum_{i=1}^{n}w_{i}r_{j}\left(t_{i}\right)}. \end{array}$$

## Appendix B

**Table A1:** Hyperparameters considered in cross-validation for random survival forest. The number of trees was set to 500.

Data	Number of Covariates Considered for Splits	Minimum Samples per Leaf
Simulation A, C	2, 4, 6	5, 10, 20, 40
Simulation B	2, 4, 7	5, 10, 20, 40
METABRIC	3, 4, 9	5, 10, 20, 40
Rotterdam/GBSG	2, 3, 7	5, 10, 20, 40

For the kernel prediction, we first considered a preliminary $\tilde{\eta }$ via cross-validation from $\left\{10^{-2},10^{-3},10^{-4}\right\}$. The final regularization parameter η was then selected via cross-validation from $\left\{\eta 2^{-3},\eta 2^{-2},\ldots ,\eta 2^{3}\right\}$. We considered fewer hyperparameter combinations for the kernel prediction than for the random survival forest.

## Appendix C

**Table A2:** Parameters of simulation B.

Degree of Non-Proportionality	$\theta_{0}$	$\theta_{1}$	$\alpha_{1}$
0	1.22	1.22	0
1	0.79	1.90	0.24
2	0.64	2.35	0.33
3	0.57	2.70	0.39
4	0.50	3.00	0.43
